# Serial intravital imaging captures dynamic and functional endothelial remodeling with single-cell resolution

**DOI:** 10.1172/jci.insight.123392

**Published:** 2021-05-24

**Authors:** Dorinne Desposito, Ina Maria Schiessl, Georgina Gyarmati, Anne Riquier-Brison, Audrey K. Izuhara, Hiroyuki Kadoya, Balint Der, Urvi Nikhil Shroff, Young-Kwon Hong, Janos Peti-Peterdi

**Affiliations:** 1 Department of Physiology and Neuroscience, Zilkha Neurogenetic Institute, and; 2 Department of Surgery, Norris Comprehensive Cancer Center, Keck School of Medicine, University of Southern California, Los Angeles, California, USA.

**Keywords:** Nephrology, Endothelial cells, Hypertension, Mouse models

## Abstract

Endothelial cells are important in the maintenance of healthy blood vessels and in the development of vascular diseases. However, the origin and dynamics of endothelial precursors and remodeling at the single-cell level have been difficult to study in vivo owing to technical limitations. Therefore, we aimed to develop a direct visual approach to track the fate and function of single endothelial cells over several days and weeks in the same vascular bed in vivo using multiphoton microscopy (MPM) of transgenic Cdh5-Confetti mice and the kidney glomerulus as a model. Individual cells of the vascular endothelial lineage were identified and tracked owing to their unique color combination, based on the random expression of cyan/green/yellow/red fluorescent proteins. Experimental hypertension, hyperglycemia, and laser-induced endothelial cell ablation rapidly increased the number of new glomerular endothelial cells that appeared in clusters of the same color, suggesting clonal cell remodeling by local precursors at the vascular pole. Furthermore, intravital MPM allowed the detection of distinct structural and functional alterations of proliferating endothelial cells. No circulating Cdh5-Confetti^+^ cells were found in the renal cortex. Moreover, the heart, lung, and kidneys showed more significant clonal endothelial cell expansion compared with the brain, pancreas, liver, and spleen. In summary, we have demonstrated that serial MPM of Cdh5-Confetti mice in vivo is a powerful technical advance to study endothelial remodeling and repair in the kidney and other organs under physiological and disease conditions.

## Graphical abstract

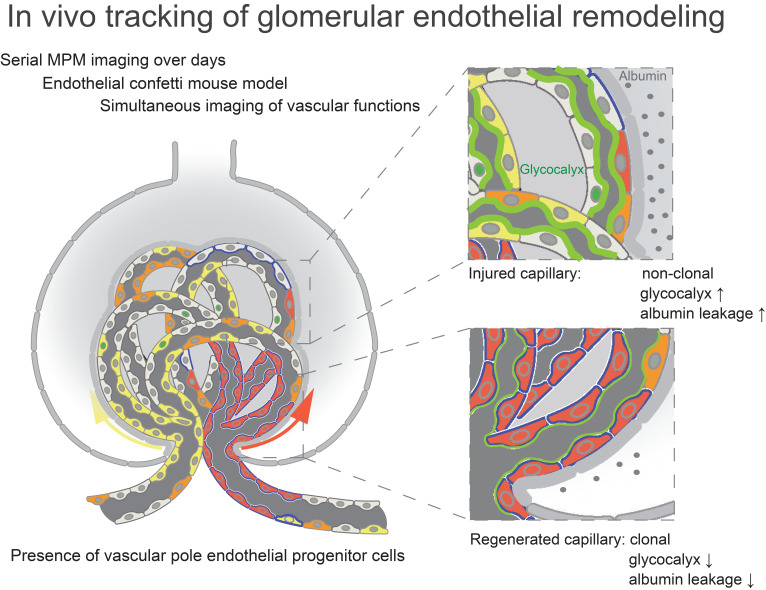

## Introduction

As in other vascular beds, endothelial cells in the kidney play important roles in the development and maintenance of specialized organ structures and functions. The uniquely flattened and fenestrated glomerular endothelial cells (GEnCs) are key players in maintaining an intact glomerular filtration barrier and the enormously high rate of plasma filtration (1–3). Numerous studies established the critical and primary role of several GEnC molecular mechanisms in angiogenesis, glomerular cell-to-endothelium crosstalk, and their alterations in the development of glomerular injury and proteinuria (2, 4, 5). These include several local, secreted factors (e.g., VEGF) and intracellular mechanisms (e.g., endothelial nitric oxide synthase–linked [eNOS-linked] pathways) that are essential in the physiological function, survival, proliferation, and differentiation of the specialized endothelium (1, 2, 5–7). Alterations in these GEnC factors can cause glomerular dysfunction, which is a common basis for the development and progression of the highly prevalent chronic kidney disease (2, 4, 5). However, despite their well-recognized role in renal physiology and kidney pathologies, the dynamics and pattern of GEnC proliferation after injury and the involvement, origin, and mechanisms of how individual endothelial precursor cells (EPCs) contribute to functional glomerular endothelial remodeling are not completely understood.

In contrast to the robust and highly complex endothelial proliferation, plasticity and vascular remodeling during vasculogenesis and angiogenesis in embryonic development (8–10), the turnover of vascular endothelial cells postnatally is considered very low in most organs, including the kidney (11, 12). Although it is known that both physiological and pathological stimuli (e.g., ischemia) can trigger angiogenesis in the adult, fundamental knowledge on the identity, origin, and role of replicative endothelial cells in systemic or renal vascular remodeling has been either lacking or controversial. The many potential sources of proliferating EPCs for renal or other organ tissue regeneration include the hematopoietic/angioblastic subpopulation of bone marrow, circulating blood cells, and local tissue-resident progenitors (13, 14). In fact, the transfer of autologous circulating EPCs can restore renal and other organ function in many pathologies, e.g., chronic experimental renovascular disease (14, 15). Importantly, the existence of local tissue EPCs in the vessel wall of adult organs is well established, and the endothelial lining of blood vessels is known to contain rare endothelial colony-forming cells that display clonal proliferative and angiogenic potential (12, 16). In the adult kidney, the contributions of both bone marrow–derived (17) and local cells (18) have been demonstrated. However, the mechanistic details and role of various EPCs in vascular remodeling and glomerular repair after injury are yet to be clarified.

A critical barrier in better understanding physiological vascular remodeling and the pathobiology of glomerular diseases has been the technical limitation to study glomerular cells, including GEnCs, in their native environment in vivo. To date, most morphological and functional observations on GEnCs were based on cell culture models in vitro and/or fixed tissue sections (1, 2, 19–22). Modern GEnC fate tracking approaches also use only cross-sectional (at 1 time point rather than dynamic) histology techniques (23, 24), which presents a technical limitation for the study of dynamic renal processes. Consequently, our knowledge on the cellular plasticity of the same glomerulus over time, the rate of turnover for individual GEnCs, and endothelial remodeling patterns in the same intact kidney in vivo are very limited. However, during the past few years, high-resolution intravital multiphoton microscopy (MPM) imaging techniques have been developed, in part by our laboratory, that allow insight into the biology of living renal cell types in the intact kidney in vivo in unprecedented detail (25, 26). MPM is a powerful noninvasive imaging technique for the deep optical sectioning of living tissues (26, 27). The basic principles, applications, advantages, and limitations of this imaging technology for the quantitative study of the living, intact kidney, including glomerular functions, have been recently described in detail (25–28). Most relevant to the present work, serial intravital MPM imaging of the same glomeruli and kidney over several days in multicolor Confetti (cyan/green/yellow/red fluorescent protein [CFP/GFP/YFP/RFP] expressing) reporter mouse models has been used recently and successfully to track the fate and function of individual renal cell types, including podocytes (29), and the cells of the renin lineage (30).

The purpose of the present study was to develop an experimental research technique to identify and track simultaneously the fate and function of single endothelial cells in vivo over time using the kidney glomerulus as a model, with the ultimate goal to improve our understanding of the mechanisms of endothelial remodeling. We demonstrate the utility of serial intravital MPM of Cdh5-Confetti mice and its applicability to other organs to directly and quantitatively visualize at the single-cell level the origin of EPCs, the dynamics, pattern, and functional consequences of glomerular endothelial remodeling in vivo in healthy and disease conditions.

## Results

### Characterization of the Cdh5-Confetti model.

Homozygous Cdh5-Confetti mice were successfully generated based on Cre-lox recombination by crossing the widely used Confetti^fl/fl^ multicolor fluorescent reporter mouse model with Cdh5-CreERT2 mice that feature tamoxifen-inducible Cre activity selectively in vascular endothelial cells. In this new transgenic mouse model, individual endothelial cells in all organs were randomly labeled after tamoxifen induction in a unique color combination (which served as an identification [ID] tag) owing to Confetti reporter construct homozygosity (2 copies per cell), and, therefore, the expression of 2 of the 4 main Confetti fluorescent proteins: membrane-targeted CFP, nuclear GFP, cytosolic YFP, or RFP. This approach provided up to 10 different potential outcomes of 2-color combinations for single-cell labeling (Supplemental Figure 1, A–C; supplemental material available online with this article; https://doi.org/10.1172/jci.insight.123392DS1). Quantitative analysis of the distribution of individual color combinations revealed that the relative abundance for RFP/GFP-, YFP/GFP-, CFP/GFP-, GFP/GFP-, YFP/RFP-, CFP/CFP-, RFP/RFP-, YFP/YFP-, CFP/YFP-, and CFP/RFP-expressing cells was 0.6% ± 0.3%, 1.3% ± 0.5%, 2% ± 0.7%, 3.6% ± 1.1%, 13.4% ± 2.5%, 14.0% ± 3.6%, 14.4% ± 1.9%, 15.6% ± 2.2%, 16.7% ± 1.7%, and 18.3% ± 2.2%, respectively, in relation to the total number of Confetti-expressing cells (a total of 296 Confetti^+^ cells were analyzed from *n* = 3 kidneys). Thus, GFP^+^ cells were rare but detectable, whereas all other color combinations were equally distributed (Supplemental Figure 1B). No transgene leakage was observed in healthy or diseased experimental mice, as confirmed by the lack of Confetti expression without tamoxifen induction. However, individual Confetti^+^ endothelial cells in randomly distributed colors could be identified in all organs studied, including the heart, kidney, liver, and brain after tamoxifen administration (Supplemental Figure 1E). Immunofluorescence staining with anti-GFP antibodies on paraffin sections of Cdh5-Confetti mice allowed us to stain CFP, YFP, and GFP^+^ cells combined in 1 color, and to perform double labeling with an endothelial cell–specific marker. Costaining for GFP and CD31 confirmed endothelial cell–specific expression of the Confetti reporter proteins (Supplemental Figure 1D). After tamoxifen induction and a 2-week washout period, Cdh5-Confetti mice did not show any renal structural or functional alterations, and had the same normal blood pressure, glomerular filtration rate, and urinary albumin excretion as WT C57Bl6 mice (data not shown).

### Tracking of endothelial proliferation and the fate of single GEnCs over several days in the same glomeruli using serial MPM.

**Figure 1:**
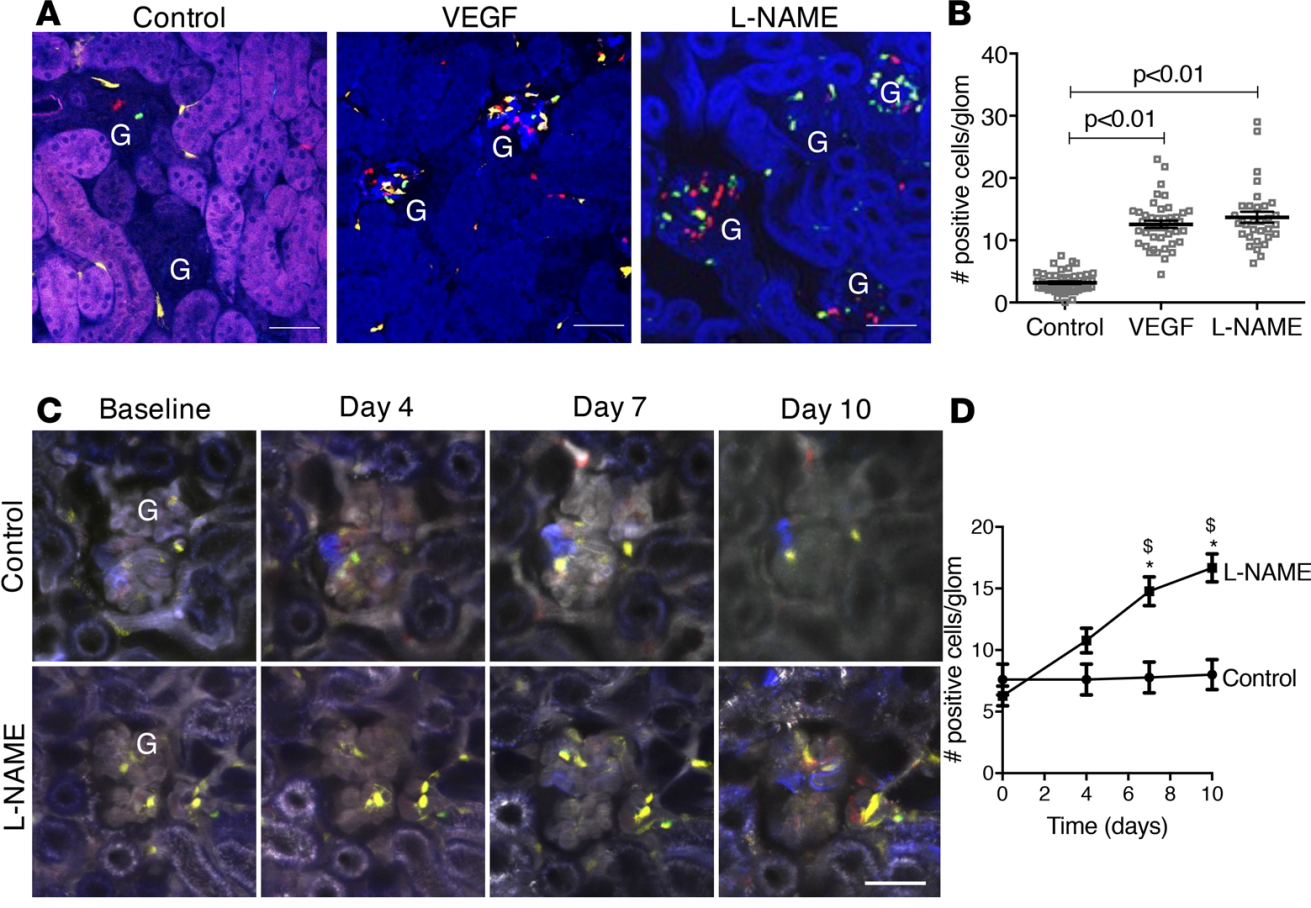
Tracking of endothelial proliferation and the fate of single glomerular endothelial cells over several days in the same glomerulus in control and in response to hypertensive injury. (**A**) Representative images of fixed frozen kidney tissue sections from Cdh5-Confetti mice demonstrating single Confetti^+^ glomerular endothelial cells (GEnCs), and the low number of Confetti^+^ cells in control, but their high density in response to VEGF (0.25 μg/injection, 1 time) or N(ω)-nitro-L-arginine methyl ester (L-NAME) treatment (1 g/L in drinking water) for 14 days. Scale bars: 100 μm (for all panels). G, glomerulus. (**B**) Summary of the number of Confetti^+^ cells per glomerulus in control, VEGF, or L-NAME–treated mice. The data are shown as the mean ± SEM, *n* = 54 (Control), *n* = 42 (VEGF), and *n* = 32 (L-NAME) glomeruli analyzed from *n* = 8–10 mice/group, using ANOVA followed by Tukey’s multiple comparison test. A *P* value of less than 0.05 was considered significant. (**C**) Single projection images of multiple optical sections (*Z*-stack) of the same glomerulus visualized by serial intravital multiphoton microscopy (MPM) over time (at baseline, days 4, 7, and 10) of a control Cdh5-Confetti mouse, and during L-NAME treatment. Plasma was labeled with i.v. injected Alexa Fluor 594–albumin converted to gray scale in the images. Scale bar: 50 μm (for all panels). (**D**) Summary of Confetti^+^ cell number per glomerulus in control versus L-NAME treatment. The data are shown as the mean ± SEM, *n* = 13 (control) and *n* = 25 (L-NAME) glomeruli tracked in *n* = 5 mice/group, using ANOVA followed by Tukey’s multiple comparison test. *P < 0.05 (considered significant versus control group); ^$^*P* < 0.05 (considered significant versus day 0 [baseline]).

We first tested the utility of the Cdh5-Confetti model for studying endothelial cell proliferation under control conditions and in response to physiological and pathological stimuli. After a single-dose partial tamoxifen induction (0.2 mg/g body weight) and 2 weeks of washout as standard protocol before all experiments below, Cdh5-Confetti mice received a single subcapsular injection of either control saline or VEGF (0.25 μg/injection). One week after treatment, mice were euthanized and kidneys were fixed and processed for histological analysis. In contrast to the low number of Confetti^+^ cells in control glomeruli, VEGF induced significant GEnC proliferation as judged by the high number of glomerular Confetti^+^ cells (VEGF, 12.6 ± 0.6 vs. control, 3.2 ± 0.2, *P* < 0.01) (Figure 1A). In addition to VEGF treatment serving as a physiological positive control, a separate group of mice received the eNOS inhibitor N(ω)-nitro-L-arginine methyl ester (L-NAME) continuously for 14 days via the drinking water (1 g/L) to test the effects of hypertensive injury. In response to L-NAME treatment, the glomerular Confetti^+^ cell number increased 4.4-fold (to 13.8 ± 0.9) compared with control (*P* < 0.01, *n* = 54 [control], *n* = 42 [VEGF], and *n* = 32 [L-NAME] glomeruli analyzed from *n* = 8–10 mice/group) (Figure 1B). The robust positive effects of VEGF and L-NAME treatment on Confetti^+^ glomerular cell number were also confirmed by 3D histological analysis of the entire kidneys using the CLARITY (Clear, Lipid-exchanged, Acrylamide-hybridized Rigid, Imaging/immunostaining compatible, Tissue hYdrogel) tissue-clearing technique and MPM imaging (Supplemental Video 1). These findings suggested rapid GEnC proliferation and glomerular vascular remodeling in response to both VEGF and hypertensive injury.

To better understand the dynamics of cellular remodeling of the glomerular endothelium in situ in the intact living kidney under control conditions and after injury, we next developed serial intravital MPM imaging of the same glomerulus in the same Cdh5-Confetti mouse and kidney over several days and weeks. By surgically implanting a dorsal abdominal imaging window (AIW) in Cdh5-Confetti mice and using very brief isoflurane anesthesia sessions subsequently, we were able to perform noninvasive MPM imaging of the same kidney at multiple time points, and to identify and track over time the position of each individual endothelial cell based on their specific color ID. On average, about 10 glomeruli were available for MPM imaging within the small kidney surface area covered by the AIW, which were all identified and marked on a map that was created to facilitate finding the same glomeruli in subsequent imaging sessions. The Confetti^+^ endothelial cell distribution of each glomeruli was registered at each time point using *Z*-sectioning of the glomerulus from top to bottom by applying the same *Z*-steps, and then the *Z*-stacks were compared in post hoc analysis. Figure 1, C and D, shows that no changes were observed in Confetti^+^ GEnC density or distribution within 10 days under control conditions, suggesting the slow dynamics of GEnC turnover in the healthy control kidney. However, in L-NAME–treated mice, serial MPM imaging of the same glomeruli found an almost 3-fold increase in the number of Confetti^+^ GEnCs within 10 days (16.7 ± 1.1) compared with baseline (6.3 ± 0.8, *n* = 13 (control) and *n* = 25 (L-NAME) glomeruli tracked in *n* = 5 mice/group), suggesting the presence of rapid GEnC proliferation and highly dynamic glomerular remodeling in response to hypertensive injury (Figure 1, C and D).

### Uniform clonal expansion of vascular pole EPCs in response to various endothelial cell injury stimuli.

To study additional features and mechanistic details of glomerular endothelial remodeling, we first applied long-term hypertensive injury in Cdh5-Confetti mice using the same L-NAME treatment model (1 g/L via the drinking water) continuously for up to 2 months. Mice developed hypertension rapidly after the beginning of L-NAME treatment, with systolic blood pressures around 140 mmHg, which stabilized after 1 month of treatment and persisted over time (Supplemental Figure 2A). Compared with baseline, when only a few Confetti^+^ cells were present, intravital MPM found multicellular tracing units in the same Confetti color in glomeruli after 15–30 days of L-NAME treatment, suggesting the clonal expansion of single EPCs (Supplemental Figure 2, B and C). By day 30 of L-NAME treatment, entire single or multiple glomerular capillary loops were continuously covered by Confetti^+^ cells of the same color (Supplemental Figure 2C). Supplemental Video 2 shows the development of several clonal GEnC clusters in multiple Confetti color combinations in Cdh5-Confetti mouse kidneys after 60 days of L-NAME treatment.

**Figure 2:**
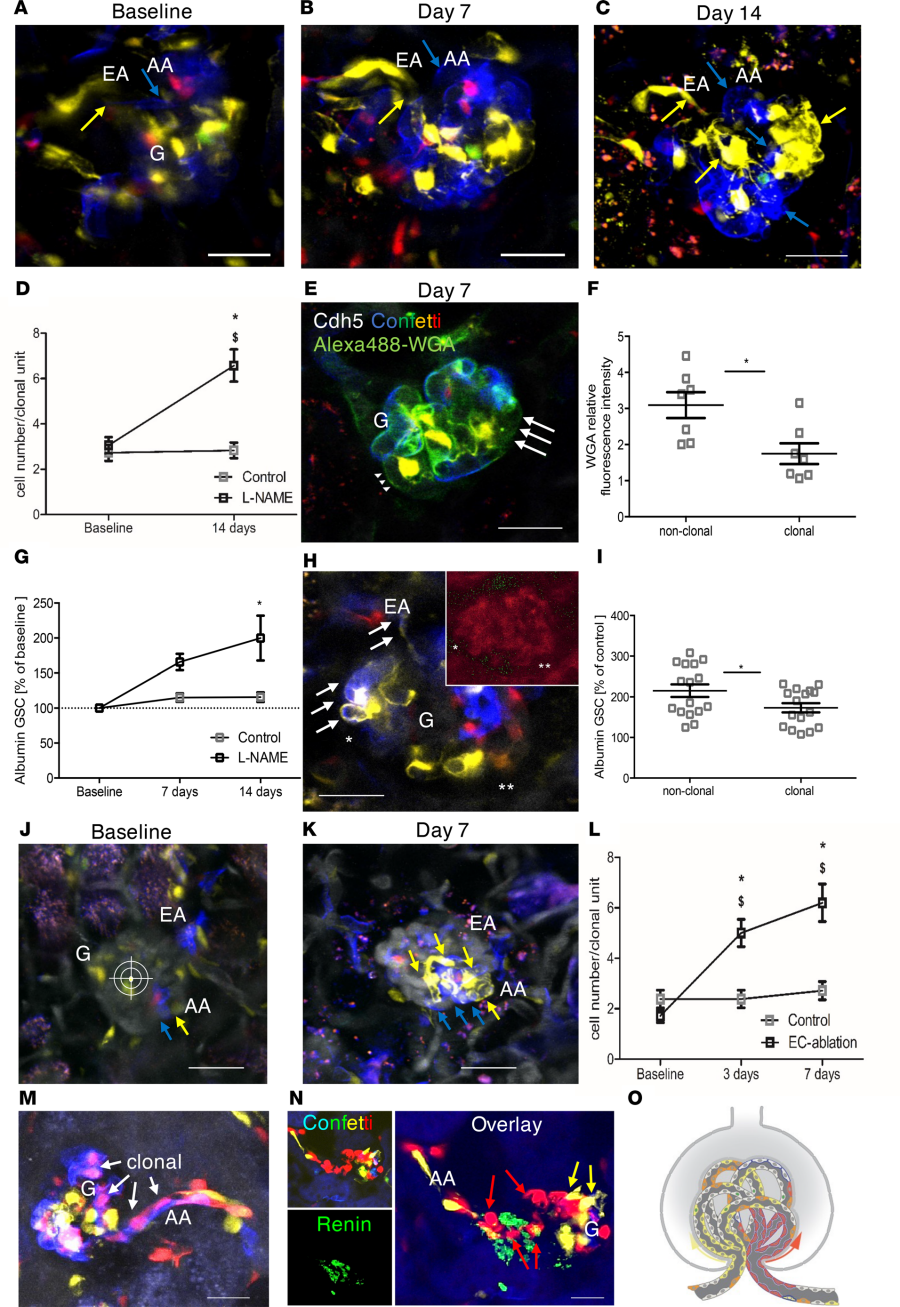
In vivo serial MPM imaging of the clonal expansion and function of local GEnC precursors. (**A**–**C**) *Z*-stack projection images of the same glomerulus at baseline (**A**) and at 7 (**B**) and 14 days (**C**) of continuous L-NAME treatment. Plasma was labeled with i.v. injected Alexa Fluor 680–albumin (gray). Arrows show blue (from afferent arteriole [AA]) and yellow (from efferent arteriole [EA]) clonal cell clusters derived from local blue/yellow EPCs in AA/EA, respectively. (**D**) Clonal cell expansion during hypertensive injury (*n* = 13, control, and *n* = 14, L-NAME glomeruli, *n* = 4–5 mice/group, using ANOVA with Tukey’s test). (**E**) In vivo staining of the endothelial glycocalyx in the same glomerulus as in **B** (Alexa488-WGA, green). Note the weak glycocalyx staining of YFP^+^ EPCs (arrows) compared with nonexpanding GEnCs (arrowheads). (**F**) Alexa Fluor 488-WGA fluorescence of nonclonal versus clonal GEnC regions (*n* = 3 mice, unpaired Student’s *t* test). (**G**) Progressive changes in albumin glomerular sieving coefficient (albumin GSC) (*n* = 6, control, and *n* = 12, L-NAME glomeruli, *n* = 5–7 mice/group, using ANOVA with Tukey’s test). (**H**) Albumin leakage (Alexa680-albumin, gray) into Bowman’s space from clonal (arrows) versus nonclonal capillary regions. Inset shows Alexa Fluor 680–albumin fluorescence with green dots equaling no signal, dark and blue dots equaling high signal. Note the higher urinary space albumin signal adjacent to nonclonal (**) compared with clonal capillaries (*). (**I**) Albumin GSC of nonclonal versus clonal GEnC areas (*n* = 6 mice, unpaired Student’s *t* test). (**J** and **K**) *Z*-stack projection images of the same glomerulus at baseline (**J**) and 7 days (**K**) after targeted laser-induced GEnC ablation. Clonal clusters derived from local yellow/blue EPCs at the vascular pole (arrows). (**L**) Clonal cell expansion after laser injury (*n* = 9, control, and *n* = 7, laser injury glomeruli, *n* = 3–4 mice/group using ANOVA with Tukey’s test). (**M**) *Z*-stack projection image of a multicolor (nonclonal) proximal AA transitioning into a clonal terminal AA and glomerulus (all cells are blue/red combination, arrows). (**N**) Renin immunofluorescence (green) with Confetti overlay confirming the terminal AA localization of clonal GEnCs (red/yellow arrows). (**O**) Schematic of EPCs localized at the glomerular vascular pole (terminal AA/EA in red/yellow, respectively) and their clonal propagation (arrows) into the glomerulus. Scale bars: 25 μm. Data are shown as the mean ± SEM. *P* < 0.05.

To test if the development of GEnC clones was linked to a specific glomerular vascular segment, it was necessary for intravital MPM imaging purposes to increase the endothelial expression of the reporter (baseline Confetti^+^ cell density). This was achieved by increasing the dose of tamoxifen (Figure 2, A–N). Taking a closer look at the clonal GEnC cell clusters, we noticed a distinct and highly reproducible anatomical pattern of Confetti^+^ cell distribution along the afferent and efferent arterioles (AAs and EAs) and glomerular capillaries in long-term L-NAME–treated mice. The proximal AA segment was always randomly multicolor (nonclonal), but transitioning into a clonal terminal AA segment (all cells were the same Confetti color combination), and then the same clone continuing into the glomerular capillaries (Figure 2M and Supplemental Video 2). Renin costaining confirmed the localization of clonal Confetti^+^ cell clusters at the terminal, renin^+^ AA segment (Figure 2N).

**Figure 3:**
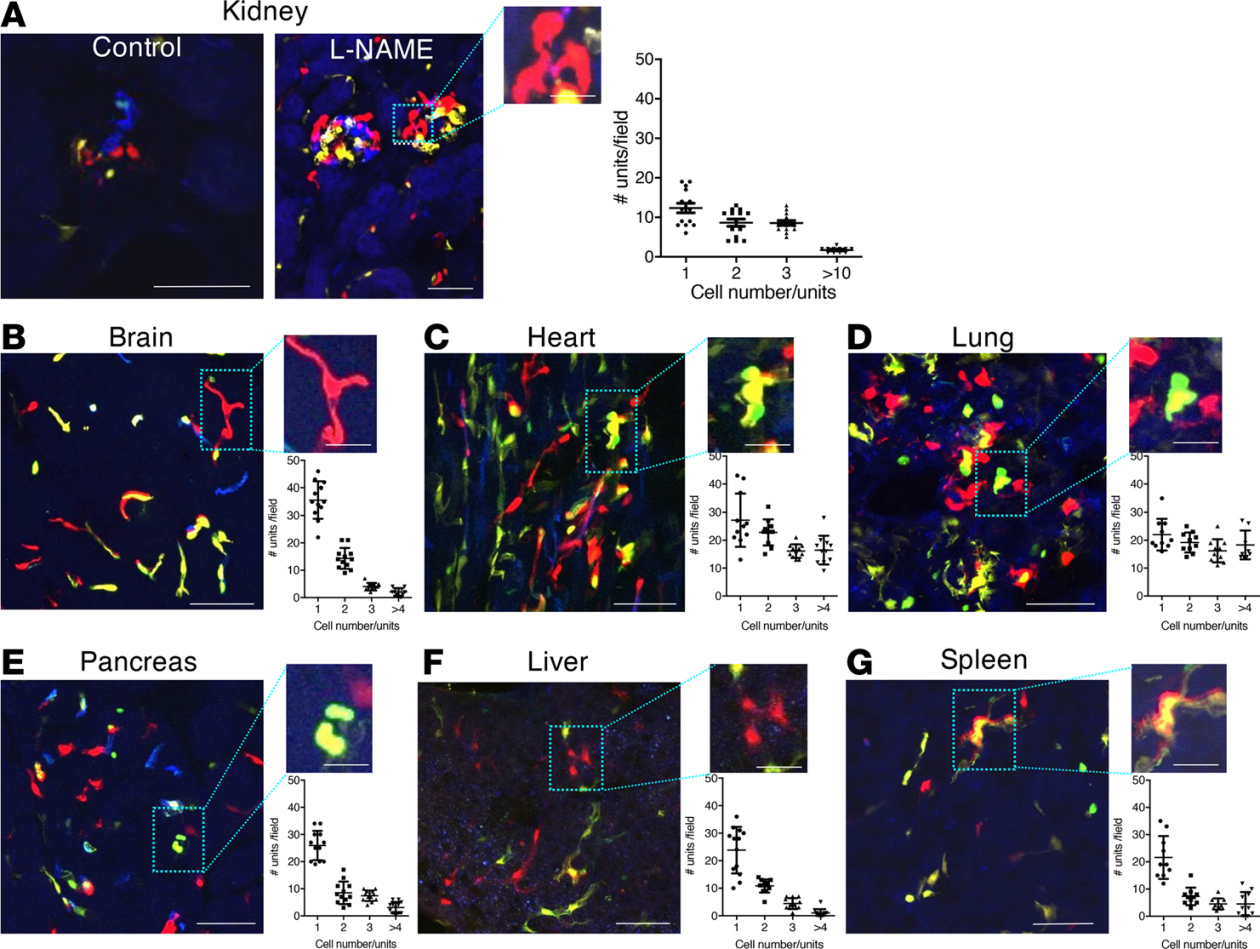
Clonal endothelial cell remodeling in different organs in response to hypertension-induced endothelial injury. (**A–G**) Representative images of fixed tissue sections from different organs (**A**, kidney; **B**, brain; **C**, heart; **D**, lung; **E**, pancreas; **F**, liver; and **G**, spleen) of Cdh5-Confetti mice after 2 months of continuous L-NAME treatment. Inset magnifications show multicellular endothelial cell tracing units appearing in the same Confetti color. Scale bars: 50 μm (for all main panels), 10 μm (for all insets). Scatter plots summarize the distribution of clonal Confetti^+^ multicell tracing units. The *x* axis shows the cell density categories (4 categories: 1, 2, 3, or more than 4 cells observed per unit) and the *y* axis shows the number of identical Confetti-colored tracing units observed per microscope field for each category. Data are shown as the mean ± SEM, *n* = 4 mice, 3–6 fields/mouse.

Serial intravital MPM imaging of the same glomeruli over several days and weeks, before and during L-NAME-induced hypertension, revealed the dynamic development of large multicellular clonal GEnC clusters at the vascular pole that appeared to originate from identical Confetti-colored cells in juxtaglomerular EA and AA segments and propagated into adjacent glomerular capillaries (Figure 2, A–D). In the same pattern, the development of clonal units at the juxtaglomerular EA/AA segments and glomerular vascular pole was observed within 2 weeks of streptozotocin-induced (STZ-induced) hyperglycemia (type I diabetes) (Supplemental Figure 3, A–D, and Supplemental Video 3). In a third injury model, we used the 2-photon laser as a micromanipulator as previously described (28, 31) to focus a point-source high-power laser beam on individual GEnCs for cell ablation in remote distances from the vascular pole. This maneuver was used to test whether endothelial cells at the vascular pole repair the injured capillary. Serial intravital MPM imaging of the same glomeruli before and 3 and 7 days after laser-induced GEnC ablation yet again revealed the rapidly developing clonal expansion of individual GEnCs in the injured capillary segment that was continuous with and matched the color of vascular pole GEnCs (Figure 2, J–L). This uniform pattern of clonal development at the glomerular vascular pole observed in 3 different injury models strongly suggests the presence, proliferation, and propagation of local EPCs at the vascular pole area (juxtaglomerular AA/EA segments) into the glomerulus (Figure 2O). Subsequent histological analysis of Confetti^+^ cell distribution on fixed Cdh5-Confetti kidney sections confirmed the presence of a very few (1–3, consisting of more than 10 cells) large multicellular clonal GEnC clusters at the vascular pole, and, in addition, some much smaller clonal GEnC clusters scattered in intraglomerular capillary regions (Figure 3A). Consistent with clonal cell expansion, immunofluorescence colabeling of Confetti and Ki67, a cell proliferation marker, identified clusters of dividing GEnCs usually at or near the glomerular vascular pole (Supplemental Figure 4). Despite the intense endothelial remodeling, no glomerular capillary aneurysms were observed in any of the applied injury models.

### Simultaneous MPM imaging of the structural and functional properties of proliferating clonal GEnC clusters.

Intravital MPM imaging uniquely allows the simultaneous assessment of renal structure and function, including the in vivo characteristics of proliferating GEnCs. Hence, we investigated clonal multicellular tracing units derived from the vascular pole for structural and functional alterations when compared with nonclonal regions, where no endothelial cell expansion was detected over time. Compared with the peritubular capillary bed, GEnCs maintain a rather thick and negatively charged endothelial surface layer, the so-called glycocalyx (3). The endothelial glycocalyx was quantitatively visualized in vivo using MPM as previously described (32), after bolus injection of fluorescent wheat germ agglutinin (WGA), which allows analysis of its density and thickness. Endothelial glycocalyx density was determined based on relative Alexa Fluor 488–WGA fluorescence intensity as previously described (32, 33) in clonal versus nonclonal capillary segments (identified from corresponding Confetti images of the same glomeruli) during L-NAME–induced hypertension. We found that capillary areas with clonal cell expansion were accompanied by a 40% reduction in endothelial glycocalyx density (Figure 2, E and F).

In addition, changes in local vascular permeability as another functional measurement of clonal GEnC clusters were assessed using MPM imaging. Continuous L-NAME treatment led to a 2-fold increase in glomerular albumin leakage compared with baseline and with time control, respectively (Figure 2G). However, when analyzing local albumin permeability of clonal versus nonclonal glomerular capillaries, we found that the albumin permeability of nonclonal capillaries was higher (215.2% ± 15.2% of control) compared with capillaries undergoing clonal remodeling (173.1% ± 11.4% of control, *n* = 16 from 6 mice in each group, *P* < 0.05).

### Endothelial remodeling in other organs.

To demonstrate the utility of the Cdh5-Confetti mouse model to study endothelial remodeling in other organs, fixed tissue sections were analyzed from the brain, heart, lung, pancreas, liver, and spleen of Cdh5-Confetti mice after low-dose induction with tamoxifen (2 mg/g body weight). Compared with the low number and even distribution of Confetti^+^ cells at baseline (Supplemental Figure 1), multicellular endothelial cell tracing units of the same Confetti color developed in all organs in response to hypertension-induced injury (2 months of L-NAME treatment, Figure 3). The presence of these multicell units in identical Confetti color suggests a local and clonal expansion of single EPCs mediating local remodeling of endothelial vasculature after endothelial injury in all organs. The highest number of multicellular tracing units was observed in the heart (Figure 3C) and lung (Figure 3D) followed by the kidney (Figure 3A), whereas the tissue sections from the brain, pancreas, liver, and spleen contained significantly fewer multicell clonal clusters (Figure 3, B, E–G, respectively). Immunofluorescence localization of Ki67 in Confetti^+^ cells on fixed tissue sections of these organs confirmed the presence of proliferating endothelial cells (Supplemental Figure 4).

### Lack of circulating Confetti^+^ EPCs in the renal vasculature.

**Figure 4:**
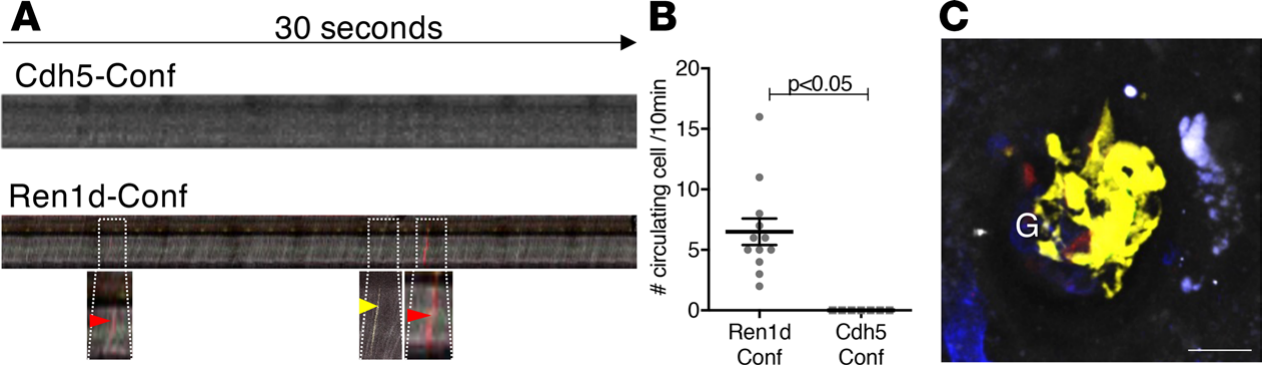
Lack of circulating Confetti+ EPCs in the intact living kidney. (**A**) Representative line (xt) scans of comparable size glomerular capillaries in Cdh5-Confetti and Ren1d-Confetti mice. Arrows in enlarged insets show circulating Confetti^+^ cells detected on line scan. (**B**) Summary of the number of circulating Confetti^+^ cells per field during 10 minutes of time lapse imaging in Ren1d-Confetti versus Cdh5-Confetti mice. Data are shown as the mean ± SEM, *n* = 3 each, 3–5 glomeruli per mouse. A P value of less than 0.05 was considered significant using ANOVA. (**C**) Single projection image of multiple optical sections (*Z*-stack) of an almost entirely clonal glomerulus (yellow Confetti^+^) visualized by intravital MPM of a control Cdh5-Confetti mouse after 60 days of continuous L-NAME treatment. Plasma was labeled with i.v. injected Alexa Fluor 594–albumin converted to gray scale. Scale bar: 25 μm. G, glomerulus.

We next tested if circulating Confetti^+^ cells can be detected in the intact living kidney of Cdh5-Confetti mice as a potential source of EPCs for local vascular remodeling. Continuous xyt time-lapse MPM imaging of large areas of the renal cortex for 30 minutes could not detect Confetti^+^ cells in the circulating blood in glomerular or peritubular capillaries (data not shown). We also performed line scans with high (1 ms) temporal resolution in glomerular capillaries to look for circulating Confetti^+^ cells. We were unable to detect any Confetti^+^ cells within glomerular capillaries, even with this highly sensitive technique (Figure 4A). In contrast, when Ren1d-Confetti mice were used as a positive control, numerous circulating Confetti^+^ cells were readily detectable in glomerular capillaries (Figure 4, A and B).

In another set of experiments, mice received multiple doses of tamoxifen (3 times total, 2 mg/g body weight each by oral gavage) for maximal induction of Confetti expression in GEnCs. After 2 months of continuous L-NAME treatment, all GEnCs remained Confetti^+^, including some glomeruli with almost entirely clonal GEnCs (Figure 4C), suggesting the lack of Confetti-circulating EPCs contributing to glomerular remodeling in response to endothelial injury (*n* = 3 mice, 10–15 glomeruli per mouse).

## Discussion

Here, we report the development and first applications of a new technical advance for experimental nephrology, vascular biology, and disease research to our knowledge. Our direct visual experimental approach used serial MPM to track, functionally characterize, and manipulate vascular remodeling by individual endothelial cells in vivo in the same vascular bed of an intact mouse organ over several days and weeks. Using the kidney glomerulus as a model, we showed that this is a unique imaging technique that is able to provide important new mechanistic details of endogenous vascular remodeling, which to our knowledge no other current technology is capable of accomplishing. These include the dynamics and pattern of the proliferation of local EPCS and clonal expansion in vivo at the single-cell level and in the exact same blood vessel, in response to physiological stimulation (VEGF administration), or during the course of disease development (hypertensive injury). Serial MPM imaging of Cdh5-Confetti mice allowed us to identify and track the fate and function of the same individually marked GEnCs. The technique provided visual clues that endothelial injury derived from 3 different types of pathologies, including hypertension, hyperglycemia, and laser-induced GEnC ablation, induced identical and highly reproducible changes. Specifically, substantial and rapid increase in the number of new GEnCs developing in clusters of the same color, beginning at the vascular pole of the glomerulus. Continuous time-lapse intravital MPM of large areas of the renal cortex with high temporal resolution found no circulating Cdh5-Confetti^+^ cells in glomerular or peritubular capillaries. Altogether, these observations suggest the presence of local EPCs at the glomerular vascular pole, and rapid glomerular endothelial cell remodeling by their clonal expansion in response to endothelial injury.

Serial MPM imaging was used recently for studying other (juxta)glomerular cell types in vivo, including podocytes and cells of the renin lineage, and provided an unparalleled view of the highly dynamic glomerular environment (29–31). However, the present work is the first study to our knowledge that utilized serial intravital MPM to image glomeruli in a completely noninvasive way using a recently developed dorsal AIW technique (34), and to track over time the fate of endothelial cells that were individually marked using multicolor fluorescent lineage tags. Advantages of imaging the exact same blood vessel in the same mouse kidney over several days and weeks using serial MPM include (a) overcoming vascular heterogeneity issues, (b) establishing the dynamics and pattern of individual cells’ proliferation and lateral propagation over time, and (c) combination with simultaneously performed functional measurements of the same blood vessel during the course of disease development (e.g., glomerular hemodynamics, albumin leakage, and endothelial glycocalyx production) (25, 32, 35). Thus, the presently applied serial MPM imaging approach uniquely revealed special structural and functional properties of proliferating GEnCs, such as rapid and clonal endothelial remodeling compared with baseline (within a few days, Figure 2, A–C, and J–L), decreased density of their endothelial surface glycocalyx and decreased albumin-permeability compared with nonproliferating GEnCs (Figure 2, E–I).

The Cre-lox–based multicolor Confetti reporter construct was previously developed (36), and has served as an excellent tool for single-cell genetic identification and fate tracking for a number of different cell types in multiple organs, including vascular endothelial cells in retina, heart, and skeletal muscle (37), and podocytes and cells of the renin lineage in the kidney (29, 30). Although the use of Confetti has been notoriously difficult with several tamoxifen-inducible CreERT2 mouse models, it worked extremely well with vascular endothelium-specific Cdh5-CreERT2 mice for unknown reasons. Even a single dose of a small amount of tamoxifen induced specific and endothelium-selective Confetti expression in a number of cells in all organs (Supplemental Figure 1B). Partial tamoxifen induction was very helpful to label, identify, and track individual GEnCs after tamoxifen washout. No new cells appeared within 10 days of timed control experiments, which confirmed the very transient nature of Cre-lox recombination and true cell fate tracking capabilities (Figure 1, A–C). In contrast to the use of heterozygous Confetti mice in most earlier studies, which can provide up to 4 different color outcomes for individual cell identification (36), the present work applied a homozygous Confetti model with up to 10 possible color combinations (Supplemental Figure 1, A and B). Therefore, the present method provided a higher degree of accuracy for single-cell identification and for determining true clonal cell expansions. In addition, the AIW technique that has been established for several internal organs, including the kidney (34), and applied in the present study was instrumental for successfully performing noninvasive serial MPM imaging at multiple time points.

Under control physiological conditions, cell fate tracking for 10 days using serial MPM of the same glomeruli did not show any change in GEnC number or distribution (Figure 1, C and D). This finding is consistent with the well-established slow physiological turnover of the endothelium in most organs, including in the kidney (11, 12). However, either the classic proangiogenic VEGF or L-NAME–induced hypertensive injury caused an almost 5-fold increase in the number of new Confetti^+^ GEnCs (Figure 1, A and B), indicating the capability of the cells for performing robust glomerular vascular remodeling under stimulated conditions. Serial MPM imaging of the same glomeruli during L-NAME treatment confirmed the appearance of new Confetti^+^ daughter cells and the high rate of GEnC proliferation within the first 10 days of treatment (Figure 1, C and D). However, constantly ongoing vascular remodeling even after these early time points was evident by the development of large, clonal (same Confetti color) GEnC clusters and occasionally entire glomeruli 1–2 months after continuous L-NAME treatment, as shown by histological analysis of fixed tissue and intravital MPM (Figure 3, Figure 4C, Supplemental Figure 2, and Supplemental Video 2). Immunolocalization of Ki-67 in several Confetti^+^ cells within glomeruli confirmed the ongoing proliferation of GEnCs in response to hypertensive injury (Supplemental Figure 4).

Detailed conventional clonal analysis of GEnC proliferation was not the purpose of the present study, since this can be performed on histological sections of fixed tissue. Nevertheless, the Confetti model in the present work behaved as a stochastic multicolor reporter with CFP/YFP/RFP color combinations consistently appearing in near-equal ratios, whereas GFP cells occurred at much lower frequencies (Supplemental Figure 1B) similarly to earlier studies (36). Serial MPM imaging visually confirmed the glomerular vascular pole as the focal origin of the most robust GEnC clonal development with short-term lineage tracing (Figure 2, A–C, J, K, and M) that progressed to monoclonality of entire capillary loops and eventually whole glomeruli by the same clone (Figure 4C, Supplemental Figure 2, B and C, and Supplemental Figure 3). These uniform results obtained from 3 different disease models suggest a hierarchical component rather than a simple stochastic model of EPC-mediated clonal endothelial remodeling of the glomerulus. In this combined model, functional heterogeneity exists within the precursor cell population with the presence of 1–3 dominant EPCs at the glomerular vascular pole, which follow a strict pattern of invariant asymmetry when they clonally remodel and propagate into the glomerulus (hierarchical component) (Figure 2O). In addition, some less potent EPCs scattered in intraglomerular capillaries form smaller clonal GEnC clusters (stochastic component). The presently identified vascular pole (juxtaglomerular) EPCs may be mature GEnCs with acquired progenitor cell characteristics, or could be a Cdh5^+^ true progenitor cell population. In either case, further studies are necessary to identify the cellular and molecular players (cell-specific markers) in their clonal expansion, e.g., the potential role of VEGF (Figure 1A), which has well-established proliferative effect on GEnCs (2) via VEGFR2, and/or angiogenic factors released from the 2 classic cell types of the juxtaglomerular apparatus (JGA), the macula densa and the renin cell. Clonal GEnC proliferation at the glomerular vascular pole under diabetic conditions found in the present study (Supplemental Figure 3) is consistent with the classic renal histopathological finding in human diabetic patients, the proliferation and growth of aberrant blood vessels at the glomerular vascular pole (38). Interestingly, renin cell–derived angiogenic factors, including VEGF, have been recently identified (39, 40). It is also known that endothelial cell fenestrations, which are typical for GEnCs, begin in the renin^+^ AA segment (41). In addition, vessel wall–resident EPCs are known to be present in other vascular beds (16). Detailed characterization of this EPC population at the glomerular vascular pole requires future work.

Importantly, the present intravital MPM imaging approach depicted not only clonal endothelial remodeling, but it was able to quantitatively visualize the functional properties of proliferating GEnCs simultaneously. These included the locally decreased production of the glomerular endothelial glycocalyx when compared with nonremodeling (nonclonal) capillary segments of the same glomerulus (Figure 2, E and F). Consistent with this finding, angiogenic peptides such as angiopoietin-2 and VEGFA/VEGFC are known to modulate the endothelial glycocalyx (42, 43) and proliferating human GEnCs show decreased proteoglycan-expression compared with nonproliferating GEnCs in vitro (44). Furthermore, disruption of the endothelial surface layer with glycocalyx-removing enzymes promoted the proliferative response of endothelial cells to shear stress (45). Overall, these data suggest that GEnCs undergo distinct alterations of their glycocalyx production and composition in vivo when proliferating. Despite the lower density of their glycocalyx, interestingly, the capillary segments newly remodeled by clonally proliferating EPCs revealed a lower vascular permeability to albumin compared with nonremodeling (nonclonal) capillary segments. This finding could be explained by a less differentiated state with only partially developed fenestrations of those cells compared with mature GEnCs. Alternatively, it could be a sign of functional regeneration of glomerular capillaries. Similarly, in the embryonic developmental stage the glomerular endothelium arises from proliferating cuboidal EPCs that initially lack fenestrations. However, during their differentiation, the thinning of their cytoplasm begins and fenestrations form (44, 46).

The utility of the Cdh5-Confetti mouse model to track vascular remodeling and clonal endothelial expansion at the single-cell level in multiple organs was demonstrated in the present experiments (for the kidney, brain, heart, lung, pancreas, liver, and spleen) and in a recent study (for the heart, retina, and skeletal muscle) (37). L-NAME–induced hypertensive injury in the present work (Figure 3) and ischemia applied in this recent study caused similar clonal expansions of endothelial cells in the heart (37), suggesting the activation of similar angiogenic programs in response to various pathological stimuli. Interestingly, after the heart and lung, the kidneys produced the highest number of multicellular clonal cell clusters in response to hypertensive injury (Figure 3), suggesting the presence of more potent angiogenic mechanisms in the kidney compared with many other organs.

Despite several experimental attempts, we could not find circulating Cdh5-Confetti^+^ cells either in control mice or in response to hypertensive injury, whereas circulating Ren1d-Confetti^+^ cells (likely bone marrow–derived B-lymphocytes; ref. 47) were readily detectable (Figure 4). This finding further emphasizes the importance of local, tissue-resident vascular EPCs in glomerular endothelial remodeling observed in the present study. Our results are consistent with recent studies arguing against the significant contribution of bone marrow–derived cells to the adult lung, liver, pancreas, heart, and kidney endothelium in control or after endothelial injury (18, 48). It should be noted, however, that mice are known to generally lack circulating EPCs compared with other species (12). In addition, the L-NAME experimental model causes not only hypertension but generalized endothelial cell dysfunction throughout the systemic circulation (49). By reducing nitric oxide bioavailability, L-NAME increases the production of pro-mitotic and chemotactic growth factors, such as HB-EGF in GEnCs (7). This effect may have further enhanced local rather than systemic EPC-mediated glomerular endothelial remodeling in the present study.

In summary, serial intravital MPM imaging of Cdh5-Confetti mice is a very useful technical advance to study the dynamics and pattern of the local vascular endothelial remodeling at the single-cell level. In contrast to conventional histological approaches, serial MPM allows quantitative visualization of endothelial remodeling from paired observations of the same vascular bed over several weeks in vivo, as well as the detection of functional alterations associated with the observed structural changes. Although the present study used the kidney glomerulus as a model, this technique can be readily applied to other organs, in which MPM is routinely performed, such as the brain, pancreas, and liver. Future use of this technology will improve our understanding of the molecular and cellular mechanisms of vascular disease and repair, the pathologies of many chronic diseases that are based on vascular dysfunction, and test the efficacy of new regenerative therapeutic approaches that target the preexisting EPCs and enhance endothelial remodeling.

## Methods

### Animals

#### Cdh5-confetti mice.

All mice used in this study were in the C57BL/6 background. Homozygous Cdh5-Confetti mice were generated by crossing mice expressing tamoxifen-inducible improved Cre recombinase under the control of the vascular endothelial cadherin Cdh5 promoter [Cdh5(PAC)-CreERT2 mice (50), originally developed by Ralf Adams, Cancer Research UK Scientist, and obtained from Cancer Research Technology Limited] and mice with the R26R-Confetti construct (36) (The Jackson Laboratory). Cdh5-Confetti mice feature vascular endothelial cell–specific expression of membrane-targeted CFP, nuclear GFP, cytosolic YFP, or cytosolic RFP.

#### Ren1d-confetti mice.

Ren1d-Confetti mice were generated by intercrossing Ren1d-Cre mice and mice with the R26R-Confetti construct as recently described (30).

### Tamoxifen induction

For fixed tissue experiments, 6-week-old mice received 0.2 mg/g body weight of Tamoxifen (MilliporeSigma) by oral gavage. For intravital imaging experiments, 3-week-old mice received 1 or 2 (on consecutive days) doses of 0.2 mg/g body weight tamoxifen by oral gavage. A 2-week washout period was granted in all induction protocols before additional procedures were performed. For MPM imaging of circulating Cdh5-Confetti^+^ cells, tamoxifen was given 3 times (1 week and 48 and 24 hours) before the first imaging session to maximize the induction of Confetti expression.

### Treatments

After the washout period, mice were randomly divided into 3 different groups, including control, L-NAME, and VEGF. L-NAME mice received the eNOS inhibitor L-NAME (1 g/L; MilliporeSigma) via drinking water continuously for 10–60 days. VEGF mice received a single-dose subcapsular injection of VEGF (0.25 μg/injection; MilliporeSigma). Briefly, animals were anesthetized with isoflurane, and the left kidney was exteriorized under sterile conditions via a small cut in the left flank. VEGF was injected using an insulin syringe (BD) inserted under the kidney capsule. After injection, the kidney was placed back into the retroperitoneum and the flank cut was closed with 2 layers of sutures, with antibiotic ointment applied. Buprenorphine-SR (0.5–1.0 mg/kg) was administered subcutaneously during the preoperative phase for analgesia. Hyperglycemia was induced in 6- to 8-week-old mice by once daily i.p. administration of 50 μg/g STZ (MilliporeSigma) for 5 consecutive days as previously described (51). In some animals, laser-induced endothelial cell ablation was performed within glomeruli of control healthy mice by focusing the laser beam that was used for fluorescence excitation on 1 or 2 endothelial cells for 5 seconds (using 100% laser power at 940 nm and 1.66 W, zoom 64, ×objective) as previously described (28).

### Serial intravital MPM imaging

A dorsal AIW above the left kidney was surgically implanted as previously described (34) that allowed completely noninvasive serial MPM imaging of the same kidney region in the same animal over time for more than 30 days. In long-term experiments (L-NAME treatment for 60 days), MPM imaging was performed on exteriorized kidneys as previously described (29). For repeated MPM imaging of same mice with AIW, animals underwent brief anesthesia sessions every 3–4 days using 1%–4% isoflurane and the SomnoSuite low-flow anesthesia system (Kent Scientific). Alexa Fluor 594– or Alexa Fluor 680–conjugated BSA was administered i.v. by retro-orbital injections to label the circulating plasma. To visualize the endothelial surface layer, 2 μg/g BW of Alexa Fluor 488–conjugated wheat germ agglutinin (Thermo Fisher Scientific) was injected i.v. as previously described (52). The animals were placed on the microscope stage, and body temperature was maintained with a homeothermic blanket system (Harvard Apparatus) as previously described (29, 30, 53). The images were acquired using a Leica TCS SP5 multiphoton confocal fluorescence imaging system with a ×40 water-immersion objective (NA 1.2) powered by a Chameleon Ultra-II MP laser at 860 nm (Coherent Inc.) and a DMI 6000 inverted microscope external nondescanned HyD detectors (Leica Microsystems Inc.). Short-pass filters (680 nm for blue and red and 700 nm for green and yellow), dichroic mirrors (cut off at 515 nm for green and yellow and at 560 nm for blue and red), and bandpass filters were specific for detecting CFP, GFP, YFP, and RFP emission (473, 514, 545, and 585 nm, respectively) (Chroma). Glomeruli *Z*-stacks were acquired with imaging settings identical to those used in the previous imaging session. The potential toxicity of laser excitation and fluorescence to the cells was minimized by using a low laser power and high scan speeds to keep total laser exposure as minimal as possible. The usual image acquisition consisted of only 1 *Z*-stack per glomerulus (approximately 3 minutes) per 3–4 days, which resulted in no apparent cell injury. *Z*-stacks from different time points were aligned using StereoMovie Maker (http://stereo.jpn.org) to visualize changes over time in a side-by-side fashion. Maximal projections from *Z*-stacks were used to count and compare Confetti^+^ cell number in the same glomerulus over time. Clonal or monochromatic tracing units were defined as numerous directly adjacent individual cells that featured the same Confetti color combination. The counting of Confetti^+^ cells and clones was facilitated by standardized image thresholding using ImageJ (NIH), Leica LAS X (Leica Microsystems Inc.), and cell-counting algorithms of Imaris 9.2 3D image visualization and analysis software (Bitplane USA) for intravital imaging *Z*-stacks.

### Blood pressure measurement

Systolic blood pressure was measured by tail-cuff plethysmography (Visitech BP-2000, Visitech System Inc.) in trained animals as previously described (54).

### Tissue processing and immunohistochemistry

After anesthesia with a combination of ketamine (100 mg per kg body weight) and xylazine (10 mg per kg body weight), animals were perfused with ice-cold PBS into the left ventricle followed by ice-cold 4% PFA for 2 minutes each, and tissues were fixed by 4% PFA at 4°C overnight. To visualize Confetti colors, tissues were embedded in OCT after sucrose cryoprotection method (30% sucrose at room temperature for 3 hours) and flash frozen. Cryosections (18 μm thickness) were imaged using the same Leica TCS SP5 microscope as previously described. Immunofluorescence staining was performed on paraffin sections (6 μm thickness). After antigen retrieval (8 minutes at 95°C in citrate buffer using pressure cooker) and blocking (30 minutes in goat blocking buffer), the sections were incubated with anti-CD31 (1:100, rabbit; Abcam ab28364) or anti-Ki67 (1:100, rabbit; Vector VP-RM04) primary antibodies followed by incubation with the secondary antibodies conjugated with Alexa Fluor 488 (1:500, anti-rabbit; Invitrogen). The same sections were then incubated with a second primary antibody anti-GFP (1:500, chicken; Aves Lab GFP-1020) followed by incubation with the secondary antibody conjugated with Alexa Fluor 594 (1:200, anti-chicken; Invitrogen). Anti-GFP antibody was used to stain CFP, YFP, and CFP components of Confetti. Confocal fluorescence microscopy was performed using the same Leica TCS SP5 microscope. Tissue clearing was performed using CLARITY.

The CLARITY tissue-clearing technique was used for 3D MPM analysis of entire Cdh5-Confetti mouse kidneys using methods as previously described (55, 56). After tissue clearing, the kidneys were set between 2 coverslips in 64% thiodiethanol and imaged using the same Leica TCS SP5 microscope as previously described.

### Statistics

Data are shown as the mean ± SEM and were analyzed in a nonblinded fashion using paired 2-tailed Student’s *t* test or 1-way ANOVA following Tukey’s multiple comparison test as indicated. A *P* value of less than 0.05 was considered significant.

### Study approval

All animal protocols were approved by the IACUC at the University of Southern California.

## Author contributions

DD, IMS, GG, and JPP contributed to the design of the study, data analysis, and the writing of the manuscript. DD, IMS, ARB, AI, HK, and YKH made substantial contributions to data acquisition. BD and UNS analyzed imaging data.

## Supplementary Material

Supplemental data

Supplemental Video 1

Supplemental Video 2

Supplemental Video 3

## Version 1 04/13/2021

In-Press Preview

## Version 2 05/24/2021

Electronic publication
