# Supplementary material for: Serial intravital imaging captures dynamic and functional endothelial remodeling with single-cell resolution
Source: JCI Insight. 2021 May 24;6(10):e123392. doi: 10.1172/jci.insight.123392 (PMC8262275; doi:10.1172/jci.insight.123392)
Supplement: Supplemental data [file jciinsight-6-123392-s138.pdf]

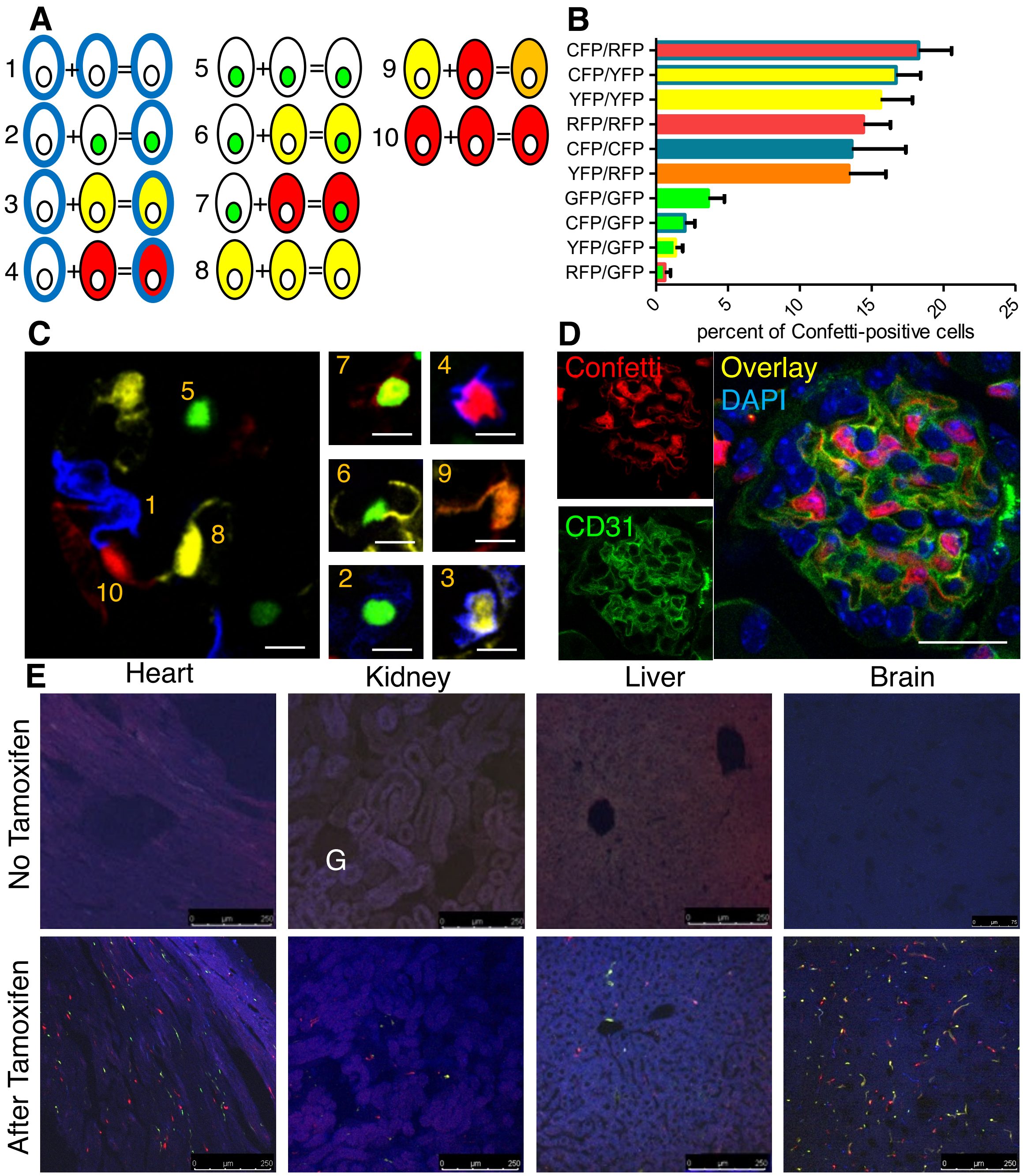

Supplemental Figure 1. Basic features of the homozygous Cdh5-Confetti mouse model.

A-C: Color combinations of single endothelial cells in the homozygous Confetti reporter model. Schematic illustration of the theoretical (A), and the actual observed quantitative color-distribution (n=296 Confetti-positive glomerular endothelial cells from n=3 mice) (B), and representative confocal microscopy images (C) of the potential color combination outcomes of Cdh5-CreERT2-mediated recombination of the Confetti reporter construct. Due to Confetti homozygosity (two copies per cell), endothelial cells were randomly labeled in one of the 10 indicated color combinations after tamoxifen induction, due to the expression of two of the four fluorescent proteins: either membrane-targeted CFP (blue), nuclear GFP (green), cytosolic YFP (yellow) or cytosolic RFP (red). **D:** Immunofluorescence co-labeling of Confetti (red) and CD31 (green) with DAPI nuclear counterstain (blue). G: glomerulus. **E:** Representative images of heart, kidney, liver, and brain fixed tissue sections of Cdh5-Confetti mice before (top) or after (bottom) tamoxifen induction. Without tamoxifen, endothelial cells were unlabeled. After tamoxifen, individual Confetti^{+} endothelial cells can be identified in all organs. Scale bars are 5 μm for all images in panel C, 20 µm for panel D and 250 µm for all images in panel E.

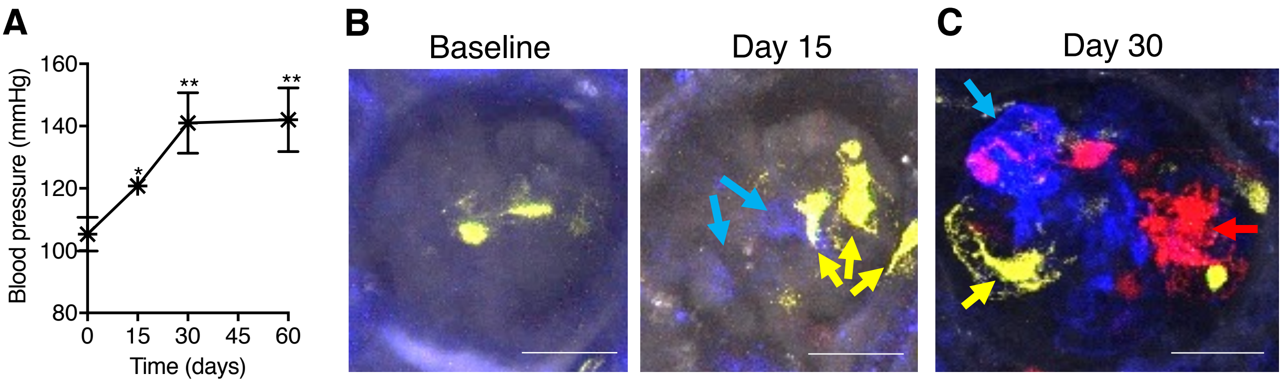

Supplemental Figure 2. Clonal expansion of local GEnC precursors in response to hypertensive injury.
A: Time-course of changes in systolic blood pressure in response to L-NAME treatment in Cdh5-Confetti mice. N=8. *: $p<0.05$, **: $p<0.01$ vs day 0, paired t-test, $P<0.05$ considered as significant. **B-C:** Representative single projection images of multiple optical sections (z-stack) of the same glomerulus visualized by serial intravital MPM at baseline and 15 days (B) and another glomerulus 30 days (C) after initiating continuous L-NAME treatment. Plasma was labeled with iv injected Alexa Fluor 594-Albumin converted to greyscale. Arrows show multi-cellular tracing units in the same Confetti color in glomeruli. Scale bar is 25 µm for all panels.

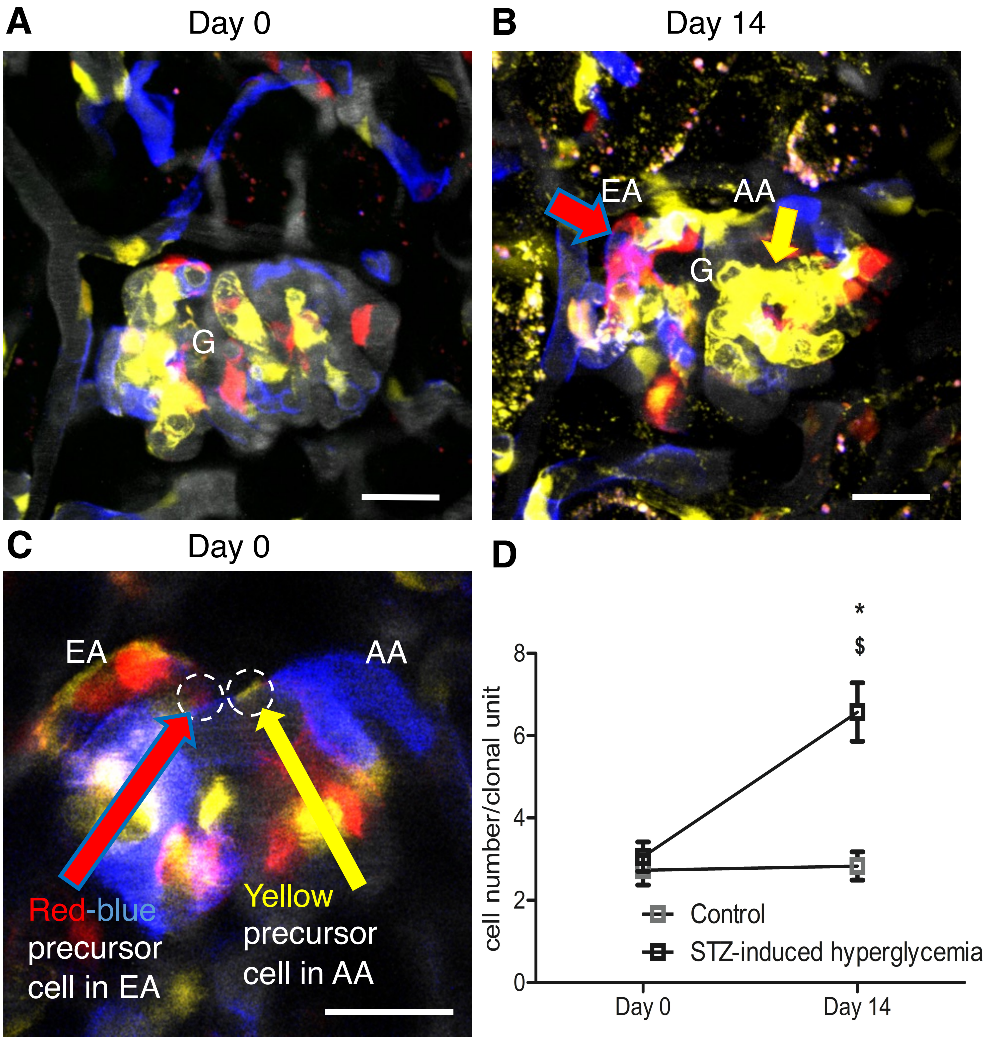

Supplemental Figure 3. Clonal expansion of local GEnC precursors in early diabetes.

A-C: Representative single projection images of multiple optical xy sections (z-stack, single xy frame series are shown in Supplemental Video 3) of the same glomerulus visualized by serial intravital MPM imaging at Day 0 (A, C) and Day 14 (B) of STZ-induced hyperglycemia (type I diabetes). Plasma was labeled with iv injected Alexa Fluor 680-Albumin converted to greyscale. Arrows show multi-cellular clonal GEnC clusters in the same Confetti color in glomerular capillaries at Day 14 (B) which were traced to begin at the terminal AA (yellow) and EA (red-blue) at the vascular pole as single GEnC precursor cells at Day 0 (arrows, C). G: glomerulus, EA: efferent arteriole, AA: afferent arteriole. Scale bars are 25 µm. **D:** Quantification of clonal cell expansion (cell number/clonal unit) during early diabetes vs. non-diabetic control. N=11 each time point/group, $^{*\$}$: $p<0.05$, vs control and Day 0, respectively, using t-test.

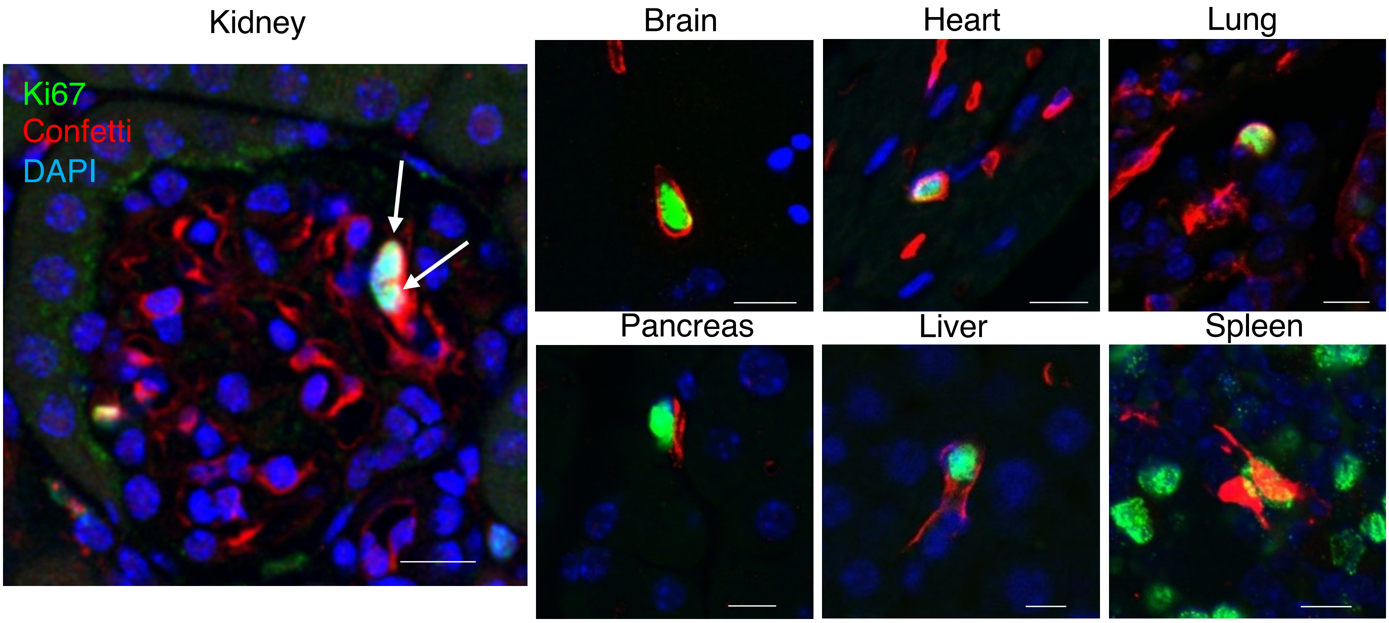

Supplemental Figure 4. Confirmation of local endothelial proliferation in different organs induced by hypertensive injury. Representative images of immunofluorescence co-staining (arrows) of Ki67 (green) and Cdh5-Confetti (red) with DAPI nuclear counterstain (blue), confirming the presence of local endothelial proliferation in response to hypertensive injury, induced by L-NAME treatment (1g/L in drinking water) for two months. Scale bars are 10 µm.

Supplemental Video 1. Optical sectioning (z-stack) of entire Cdh5-Confetti kidneys after tissue clearing using CLARITY. Representative z-stacks obtained by MPM imaging of optically cleared entire Cdh5-Confetti kidneys from control, and after VEGF and L-NAME treatment. In contrast to the low number of Confetti$^+$ cells in control, a significant increase in Confetti$^+$ cell number is visible in response to VEGF and L-NAME treatment. Scale bar is 100 µm for all panels. Optical z-sectioning was performed in 1.5 µm steps. Starting from the kidney surface, the initial 300 µm thick volume of renal cortex is shown. Non-specific autofluorescence (greyscale) is included to better illustrate tissue microanatomy.

Supplemental Video 2. Optical sectioning (z-stack) of long-term L-NAME treated Cdh5-Confetti kidneys *in vivo* using MPM imaging. Representative z-stacks obtained by intravital MPM imaging of Cdh5-Confetti kidneys after L-NAME treatment for 60 days. Note the appearance of multi-cellular tracing units in the same Confetti-color combination (yellow, blue/red, blue, and green/yellow examples are shown) starting in the terminal afferent arteriole segment and continuing into glomerular capillaries, suggesting the clonal expansion of local EPCs. Plasma was labeled with Alexa Fluor 594-Albumin and appears in greyscale. Scale bar is 100 µm for both panels. A 60 µm thick volume of renal cortex is shown in 2 µm steps.

Supplemental Video 3. Optical sectioning (z-stack) of the same glomerulus in a Cdh5-Confetti mouse kidney visualized by serial intravital MPM imaging at Day 0 and Day 14 of STZ-induced diabetes. Note the appearance of multi-cellular tracing units in the same Confetti-color combination starting in both the terminal efferent arteriole (red/blue) and afferent arteriole segment (yellow) and continuing into glomerular capillaries, suggesting the clonal expansion of local EPCs. Plasma was labeled with Alexa Fluor 594-Albumin and appears in greyscale. Scale bar is 100 µm for both panels. A 60 µm thick volume of renal cortex is shown in 2 µm steps.
